# The continuing value of mesalazine as first-line therapy for patients with moderately active ulcerative colitis

**DOI:** 10.3389/fgstr.2024.1335380

**Published:** 2024-06-03

**Authors:** Kristine Paridaens, Matthew J. Freddi, Simon P. L. Travis

**Affiliations:** ^1^ Medical Affairs, Ferring International Center, St-Prex, Switzerland; ^2^ Health Economics and Outcomes Research (HEOR), Violicom Medical Limited, Reading, United Kingdom; ^3^ NIHR Oxford Biomedical Research Centre, Oxford University Hospitals NHS Foundation Trust, John Radcliffe Hospital, Oxford, United Kingdom

**Keywords:** aminosalicylates, mesalamine, 5-ASA, inflammatory bowel disease, delayed-release

## Abstract

Mesalazine is an established and recommended first-line treatment for mild-to-moderate ulcerative colitis (UC). For patients with moderately active UC, the choice to use mesalazine or to initiate treatment with an oral corticosteroid or anti-tumor necrosis factor (TNF) agent is not clearly informed from current guidelines. The use of mesalazine is supported by robust clinical evidence supporting its efficacy at inducing remission in patients with moderately active disease. A key advantage of mesalazine is its tolerability profile being similar to that of placebo, which contrasts with that of the corticosteroids and advanced therapies, where there is the potential for significant toxicities. Mesalazine also has cost advantages over anti-TNFs and other advanced therapies. Evidence supports the consideration of all patients with moderately active UC for first-line mesalazine therapy at an optimized dose of ≥4g/d (± 1g/d rectal). Patients responding to treatment within 2 weeks should continue at ≥4g/d for at least 6 months before a dose reduction is considered, since this then alters the pattern of disease.

## 1 Introduction

Mesalazine, also known as mesalamine or 5-aminosalicylic acid (5-ASA), has been a mainstay of treatment for mild-to-moderate ulcerative colitis (UC) for over three decades. Current clinical guidelines, such as those from the European Crohn´s and Colitis Organisation (ECCO) and American Gastroenterological Association (AGA), recommend mesalazine as first-line treatment of mild-to-moderately active UC (1, 2). These same guidelines also provide recommendations on moderate-to-severely active UC, where oral corticosteroids and anti-tumor necrosis factor (TNF) agents are standard initial therapies (1, 2). When considered together, these recommendations leave it unclear what should be the most appropriate first-line treatment for patients with moderately active UC, particularly as there is no universally applied definition of disease severity. Indeed, the ECCO guidelines state that: “*We recognise that these divisions [mildly-to-moderately active disease and moderately-to-severely active disease] are somewhat arbitrary, partially overlapping, and inconsistently defined*…” (1).

To aid clinical decision-making, we present the rationale and evidence supporting the continuing role of mesalazine as first-line treatment of moderately active UC.

## 2 Ulcerative colitis and defining moderately active disease

UC is a chronic inflammatory condition affecting the intestinal mucosa of the colon and rectum, characterized by periods of active disease interspersed by periods of remission (3). In active UC, the most common symptoms exhibited are bloody diarrhea with urgency, increased frequency and abdominal pain. Additional symptoms include fatigue, weight loss, and may include extra-intestinal manifestations, such as anemia, joint inflammation, or mouth ulcers (4). The symptoms of UC can lead to a profound negative impact on multiple aspects of patient quality of life (QoL), including functional, psychological, social and occupational (5, 6). These detrimental impacts on QoL correlate with increasing disease severity (6).

A number of clinical and endoscopic assessment tools exist for the assessment of UC severity, including Truelove & Witts’ criteria, the Mayo Clinic score, the UC Disease Activity Index (UCDAI), Simple Clinical Colitis Activity Index (SCCAI), and the UC Endoscopic Index of Severity (UCEIS) (7–11). However, there is no consensus on the most appropriate tool to use across guidelines or within clinical practice (2, 4, 9, 12). Moderately active UC is typically defined as a stool frequency (SF) of >4 times daily with urgency and visible blood, but without systemic features (8).

## 3 Evidence and rationale for mesalazine in moderately active ulcerative colitis

The greater part of clinical evidence for mesalazine has focused on it use across the spectrum of mild-to-moderate UC, with Cochrane reviews (13, 14) and other meta-analyses (15, 16) confirming its effectiveness at inducing and maintaining remission in patients with extensive, left-sided, or distal disease. Several studies and a recent meta-analysis have assessed the use of mesalazine specifically in patients with moderately active UC (17–20).

In a study by Kamm et al. (17) of MMX mesalazine 2.4g/d and 4.8g/d, both doses achieved similarly higher rates of combined clinical and endoscopic remission (UCDAI score ≤1, rectal bleeding [RB]=0, SF=0, ≥1 point reduction from baseline in sigmoidoscopy score at week 8) than placebo (38.9% and 36.0% *vs* 22.8%, respectively; p-value not reported) in patients with moderately active UC (modified UCDAI score 6–10). The study also included 2.4g/d Eudragit-S-coated mesalazine as a reference arm, which had a combined remission rate of 30.2% (17). In contrast to this study, results from the ASCEND II and III studies suggested an advantage when using higher doses of mesalazine (18, 19). In patients with moderately active UC (Physician’s Global Assessment [PGA]=2 points, SF=1, RB=1, ≥2 points in sigmoidoscopy assessment with positive friability assessment), increased rates of treatment success (overall improvement at week 6) were achieved with Eudragit-S-coated mesalazine 4.8g/d than 2.4g/d (ASCEND II: 71.8% *vs* 59.2%, respectively, p=0.036; ASCEND III: 70.2% *vs* 65.5%, p=0.17). Rates of clinical (SF=0 and RB=0) and complete remission (no clinical evidence of disease and normal endoscopy) also tended to be higher for the 4.8g/dose, albeit reaching statistical significance only for the former (clinical: 43% *vs* 35% for 2.4g/d, p=0.04; complete: 20.2% *vs* 17.7% for 2.4g/d, p-value not reported) (18, 19). Of clinical relevance, the time to cessation of RB was significantly shorter in patients who received 4.8g/day compared with those on 2.4 g/day (9 *vs* 16 days, respectively; p=0.035) (18). The clinical relevance of this is that if rectal bleeding has not stopped within 10 days on high dose (>4g) mesalazine, then the patient will be a slow or incomplete responder, so treatment can be escalated at that stage.

More recently, a network meta-analysis only in patients with moderately active UC, used data from the Kamm et al. (17) and ASCEND II & III studies (18, 19). This reported that oral prolonged-release mesalazine 4g/d has broadly similar efficacy at inducing combined clinical and endoscopic remission to MMX mesalazine 4.8g/d and Eudragit-S-coated mesalazine 4.8g/d (20). Whilst there was no statistical difference between oral prolonged-release 4g/d and MMX 4.8g/d, there was an approximately 5% difference between oral prolonged-release 4g/d and Eudragit-S-coated mesalazine 4.8g/d in favor of the former (20).

All these studies included patients with left-sided or extensive disease. In patients with moderately active distal disease, the real-world QUARTZ study found that 75.6% and 82.4% of those treated with oral and/or rectal prolonged-release mesalazine were in clinical remission (Mayo clinical subscore [excluding endoscopy] ≤2 with no item >1) at 8 weeks and 12 months, respectively (21). Corresponding results for normal or inactive disease at endoscopy (Mayo endoscopy score <1) were 57.1% at 8 weeks and 61.5% at 12 months (21). The study also assessed the important outcome of health-related QoL (Short Inflammatory Bowel Disease Questionnaire) and this was found to be significantly improved in patients with moderately active disease receiving prolonged-release mesalazine (total score 43.3 at week 8 *vs* 35.9 at baseline; p<0.001) (21). This 7.4 point difference in scores did not quite reach what is generally considered a clinically meaningful change of 9 points, but was achieved in a patient population with distal disease and a comparatively low baseline QoL (35.9 *versus*, for example, 44.9 in the EpiCom cohort) (21, 22).

In addition to its proven efficacy, a strong rationale for mesalazine use is its excellent tolerability profile. Different meta-analyses have reported similar adverse event (AE) results for mesalazine and placebo (13, 15). The most commonly reported adverse events associated with mesalazine are headache, nausea, abdominal pain, nasopharyngitis, rash, loss of appetite, flatulence and fever (13). Nevertheless, these events are as common in patients receiving placebo in the randomized controlled trials. The lack of notable AEs is likely in large part due to the topical effect of mesalazine on the intestinal mucosa, since mesalazine acts on, is metabolized by and excreted from intestinal epithelial cells. Unusually for medications with documented efficacy, studies have found no clinically relevant difference in AE profile or rate with higher (>2g) *versus* lower (<2g) mesalazine doses (15, 17–19).

Alternative treatments for moderately active UC, including oral systemic corticosteroids and advanced therapies (*e.g.* adalimumab, filgotinib, golimumab, infliximab, tofacitinib, ustekinumab and vedolizumab), are effective and generally well-tolerated (23–26). Nonetheless, salient limitations to their use is their potential for debilitating and serious AEs, particularly with longer-term use. The side effects of systemic corticosteroids are well recognized, including infections, psychological disturbances, weight gain, gastrointestinal (GI) AEs, hirsutism, alopecia, vertigo, venous thromboembolism (VTE), cardiovascular disease, myopathy and osteoporosis (27–30). Although no guidelines recommend the long-term use of corticosteroids, it is recognized that approximately 5–15% of patients with UC may be on chronic steroidal treatment (defined as >3 or 6 months therapy) in clinical practice (31, 32), albeit it would be expected that the majority would have disease at the severer end of the spectrum. Advanced therapies also have the potential to cause AEs such as injection site reactions (ISRs)/infusion-related reactions, serious infections, behavioral disturbances, hypertension, anemia and musculoskeletal disorders, while some increase the risk of malignancy (*e.g.* lymphoma), cardiovascular events, VTE and serious skin conditions (23, 25, 26). When considering severe UC (≥6 bloody stools *per* day with additional signs of toxicity (8)) the balance would lean towards the efficacy of advanced therapies exceeding the potential risks of toxicity. However, for patients with moderately active disease, the potential for serious AEs assumes increasing importance, particularly when there is the option to initiate mesalazine treatment with its efficacy and safety in this population. From a healthcare provider perspective, the treatment costs of mesalazine are also advantageous, being lower than that of advanced therapies (33).

## 4 Quantifying the benefits of mesalazine in moderately active ulcerative colitis

To illustrate the potential clinical and cost benefits of mesalazine for patients with moderately active UC, a modeling exercise was undertaken. This used a modified version of a published model that quantified the benefits of optimized mesalazine treatment strategy across the spectrum of mild-to-moderate disease (34). The modeling focused on the induction of remission in patients with moderately active disease and compared two treatment strategies (**Supplementary Figure 1**):

1. High-dose mesalazine (≥4g/d oral, plus budesonide MMX in left-sided or extensive disease as included in the previous model (34)) as first-line treatment before oral systemic steroids and anti-TNFs; *versus*,
2. Oral systemic steroids as first-line treatment before anti-TNFs.

The model compared outcomes for a hypothetical population of 10,000 patients who were followed until remission or requirement for anti-TNF treatment (*i.e.* maintenance treatment was not modeled). Efficacy inputs were derived from the available literature and, data permitting, focused on combined clinical and endoscopic remission (**Table 1**) (20, 35, 36, 43). Patients are assumed to be equally adherent in both model arms and to be equivalent in adherence to that seen in clinical data. Remission rates for mesalazine were extracted from the most recent meta-analysis in moderate UC (20). The efficacy for budesonide MMX was extracted from the combined analysis of the CORE I and CORE II trials (44, 45), which included a subgroup analysis of patients with moderate UC (35). For systemic steroids, a meta-analysis that included both beclomethasone dipropionate and prednisolone was used (36), albeit the data for the latter were derived from a single study (23). Mesalazine and budesonide MMX were assumed to cause no major AEs (as outlined above) (15, 35), while several notable AEs for systemic corticosteroids and anti-TNFs were assessed (27, 39–42). Treatment acquisition costs were taken from the British National Formulary (October 2023) (33) and administration costs from the UK National Health Service (NHS) reference costs (2021–2022) (37) or Personal Social Services Research Unit data (2022) (38). The benefits of mesalazine as first-line treatment were expressed as the number of patients avoiding systemic corticosteroids and anti-TNF therapy due to remission being achieved without the need to escalate to these therapies. In addition, the potential AEs avoided and cost savings related to this reduced use were also calculated.

**Table 1:** Model inputs.

Treatment	Dose	Remission rate (20, 23, 35, 36)	Cost (33, 37, 38)^◊^	AE rate (27, 39–42)
Mesalazine	≥4g/d oral	25.7%*	£243.66^^^	Assumed no significant
Budesonide MMX	9mg/d	14.1%^†^	£140.00	Assumed no significant
Beclomethasone dipropionate	5mg/d	34.8%^‡^	£5.58	GI: 0.13% Neurological: 0.11% Dermatological: 0.08% Psychological: 0.05% Infections: 0.04% VTE: 0.004%
Prednisolone	40–30mg/d	21.5%^§^		
Anti-TNFs (adalimumab [A], infliximab [I], golimumab [G])	A: 160/80/40mg 14d SC I: 5mg/kg at w0, w2 and w6 IV G: 200/100mg 14d SC	Not included in model	£4,081.48^^^	ISRs: 3.3–6.3% Infusion-related reactions: 11.6% Serious infections: 0.3–0.6% Pyrexia: 1.8% Anaemia: 2.7%

^◊^Cost for course of treatment (oral corticosteroids: 4 weeks; golimumab: 6 weeks; mesalazine/budesonide MMX/adalimumab/infliximab: 8 weeks). ^^^Average cost across all products. *Data used in meta-analysis: combined remission defined as a Clinical and Endoscopic Response Score of rectal bleeding scores of 0 and stool frequency score of 0 or 1 with at least 1 point decrease from baseline, with an endoscopic score of 0 or 1; remission defined as a modified UCDAI score of ≤1 with a score of 0 for rectal bleeding and stool frequency and at least a 1-point reduction from baseline in sigmoidoscopy score; remission defined as normal stool frequency, no rectal bleeding, patient’s functional assessment score of 0, normal endoscopy findings and PGA score 0 assessment); remission defined as PGA score of 0 (i.e. complete resolution or normalization of stool frequency, rectal bleeding, and sigmoidoscopy with contact friability assessment score.

^†^Combined remission defined as UCDAI score of ≤1, with no rectal bleeding (UCDAI subscore=0), normal stool frequency (UCDAI subscore=0), normal mucosa with no evidence of friability at full colonoscopy and an endoscopic index score ≥1 point lower than baseline. ^‡^Figure derived from meta-analysis: clinical remission defined as DAI score ≤1 and an endoscopically documented mucosal healing; DAI score <3; DAI score <1. ^§^Endoscopic remission defined as endoscopic score of 0 on DAI. AE, adverse event; DAI, Disease Activity Index; GI, gastrointestinal; ISR, injection site reaction; IV, intravenous; PGA, Physician’s Global Assessment; SC, subcutaneous; UCDAI, Ulcerative Colitis DAI; VTE, venous thromboembolism.

**Figure 1:**
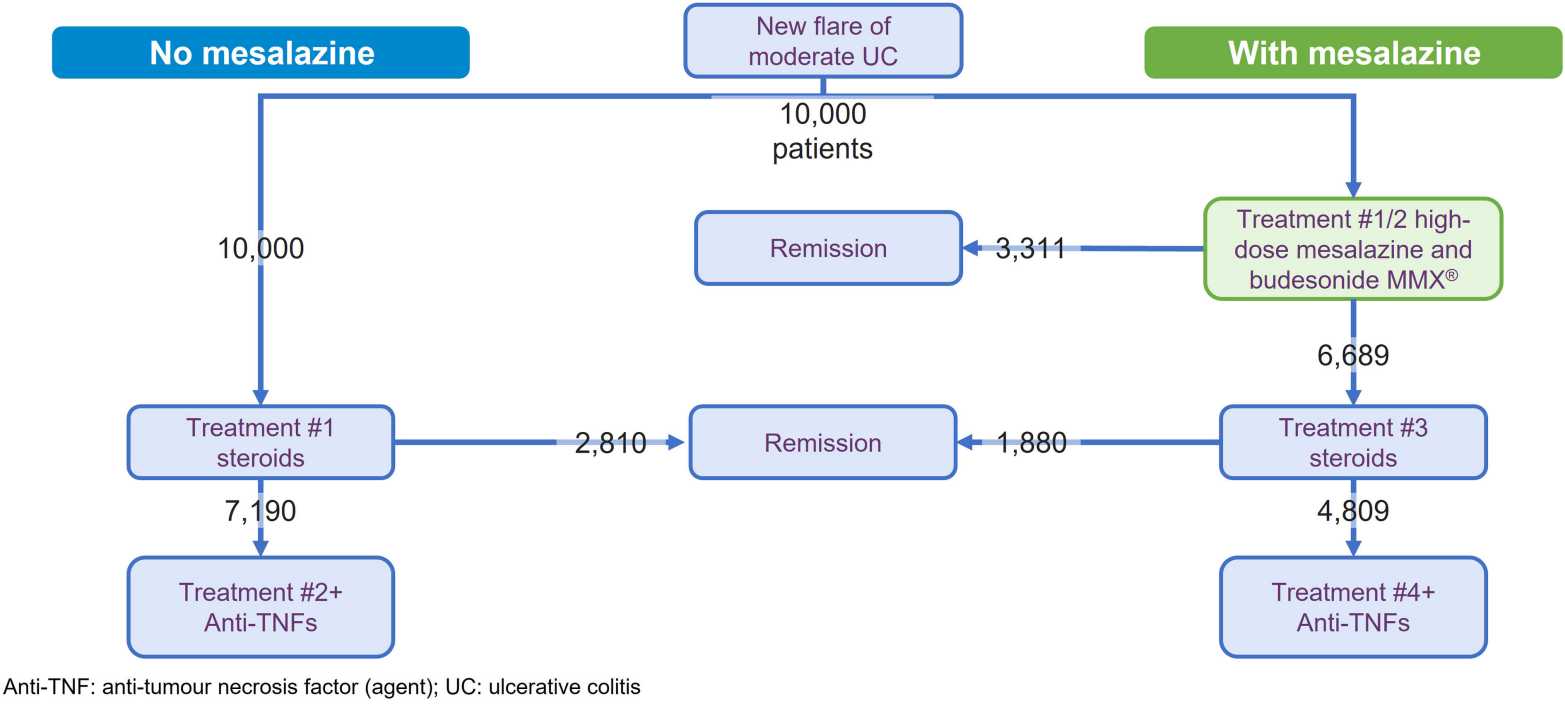
Results of model comparing the first-line treatment with and without mesalazine treatment in the induction of remission for moderately active ulcerative colitis.

The model projected that the use of mesalazine as first-line therapy was associated with 3,311 fewer patients (33.1% of modeled patient population, **Figure 1**) requiring systemic corticosteroids (relative reduction: 33.1%) and 2,381 (23.8%) requiring anti-TNFs (relative reduction: 33.1%). An alternative visualization of these results, using 100 patients as the input population, is included as **Supplementary Figure 2** as this may be more intuitive for some readers. The model calculated that this reduction in exposure to systemic corticosteroids resulted in the potential to avoid up to 430 GI AEs, 357 neurological AEs, 273 dermatological AEs, 166 psychological AEs, 148 infections, and 12 VTEs. With the reduction in use of anti-TNF agents, up to 150 ISRs or 276 infusion-related reactions, 14 serious infections, 64 cases of anemia, and 43 cases of pyrexia would potentially be avoided. Whilst no AEs for mesalazine (or budesonide MMX) were included in this analysis, the toxicities highlighted for the systemic corticosteroids and anti-TNFs were far from exhaustive due to limitations in the data available.

Financially, the use of mesalazine was associated with an overall saving of £6,565,382 *versus* initiating treatment with systemic corticosteroids and progressing to anti-TNFs (saving of £9,736,138 with costs of mesalazine/budesonide MMX treatment of £3,170,756). This equated to a *per* patient saving of about £656. Additional breakdown by mesalazine formulation found cost savings of £688 *per* patient for prolonged-release mesalazine, £648 for MMX mesalazine, and £610 for Eudragit S coated mesalazine (**Supplementary Table 1**), predicated on the efficacy reported in the meta-analysis (remission rate: 34.1%, 32.9% and 31.6%, respectively) (20).

A key limitation of the modeling was the variability in the definition of remission across the studies. This was particularly striking for systemic corticosteroids and budesonide MMX, where the latter used the strictest definition of remission (combined clinical and endoscopic remission, where RB=0 and SF=0, with normal mucosal and no friability on full colonoscopy, and a ≥1 point reduction in endoscopy score, meaning a DAI <1). In contrast studies of systemic steroids defined remission as a DAI score ≤3 ± mucosal healing (22, 43–45). Assuming that stricter assessment criteria would result in decreased remission rates, a sensitivity analysis was performed reducing the efficacy of the systemic corticosteroids by a nominal 10% or 25%. The number of patients avoiding anti-TNFs was 2,473 with a 10% rate and 2,612 with a 25% rate (*versus* 2,381 in the base case). This corresponded to a cost saving *per* patient of £694 and £751, respectively (base case: £656.54). As the use of mesalazine leads to fewer patients requiring steroids, the reduction in the steroidal remission rate meant that more patients in the no mesalazine treatment pathway were affected by this change, thus increasing the differential benefit for mesalazine. It is also acknowledged that there is a very small but definite risk of serious AEs with mesalazine, such as interstitial nephritis, nephrotic syndrome, or pneumonitis. Such events remain the subject of case reports, so their inclusion in the model, even with a hypothetical population of 10,000, would not be expected materially to affect the results.

## 5 Discussion

The evidence presented demonstrates that mesalazine has a well-supported history as effective and well-tolerated first-line therapy for patients with moderately active UC. Use of mesalazine should be considered for all patients with moderately active UC, particularly those with newly diagnosed disease or who have relapsed after a long period of remission. An optimized oral dose of mesalazine ≥4g/day (± 1g/d rectal mesalazine for proctitis) is strongly recommended to maximize the chances of treatment success. For patients not responding to mesalazine therapy (*e.g.* no reduction or cessation of RB within 2 weeks), then escalation to oral corticosteroids or advanced therapies should be explored. For those who do respond to mesalazine, however, the IMPACT study has shown that longer durations of treatment for at least 6 months with mesalazine 4g/d led to a significant reduction in the risk of relapse (46). If a dose reduction is considered following a sustained period of remission, then guidelines recommend that an oral dose of at least 2g/day mesalazine should be maintained (1, 2).

The advantages of using mesalazine as first-line therapy include the avoidance of potentially serious AEs associated with systemic steroids and the advanced therapies, as well as cost savings to the healthcare system, as supported by the modeling. In addition, avoiding the use of anti-TNFs means that if they are required subsequently in the disease course, then the potential issue of immunogenicity (antidrug antibodies) compromising efficacy will be deferred (42, 47). Likewise, the cumulative effects of steroids on, for example, bone density, would be minimized since fewer courses would need to be prescribed (27). When considering long-term maintenance therapy, the cost savings of mesalazine in preference to the anti-TNFs would be considerable. It is clear that some patients with moderately active UC will need steroids and/or advanced therapies. The point is that some – perhaps many – do not. Such patients matter.

In view of an increasing worldwide prevalence of UC, particularly in less industrialized countries (48–50) where restrictions on healthcare budgets are even greater than Western Europe or North America, it is important that the therapeutic armamentarium for UC be used effectively. Optimal mesalazine therapy remains the starting point of treatment for patients with moderately active UC.

## Data availability statement

The raw data supporting the conclusions of this article will be made available by the authors, without undue reservation.

## Author contributions

MF: Data curation, Formal analysis, Funding acquisition, Investigation, Methodology, Resources, Validation, Visualization, Writing – original draft, Writing – review & editing. KP: Conceptualization, Data curation, Formal analysis, Funding acquisition, Investigation, Methodology, Project administration, Resources, Supervision, Validation, Visualization, Writing – original draft, Writing – review & editing. ST: Conceptualization, Funding acquisition, Investigation, Methodology, Supervision, Validation, Writing – original draft, Writing – review & editing.
