# Supplementary material for: The continuing value of mesalazine as first-line therapy for patients with moderately active ulcerative colitis
Source: Front Gastroenterol (Lausanne). 2024 Jun 3;3:1335380. doi: 10.3389/fgstr.2024.1335380 (PMC12952326; doi:10.3389/fgstr.2024.1335380)
Supplement: Supplementary file 1 [file DataSheet_1.docx]

Supplementary Material

# Supplementary Figures and Tables

## Supplementary Figures


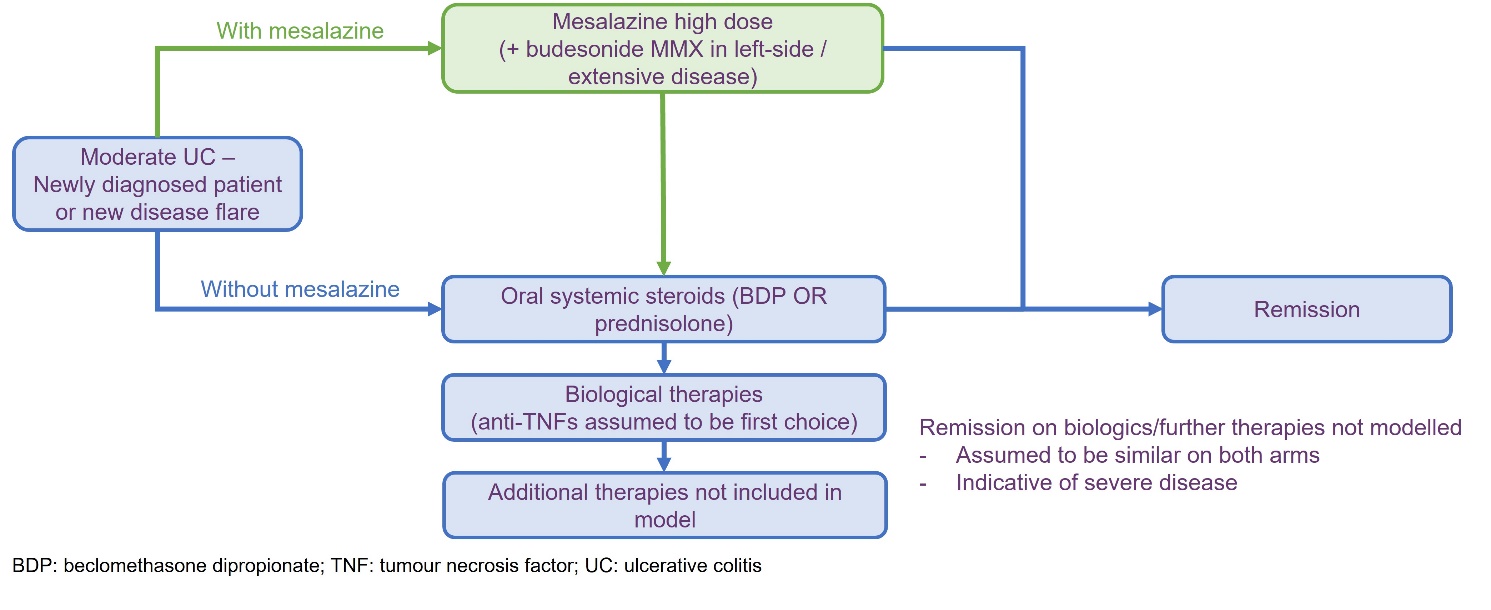


**Supplementary Figure 1.** Diagram of the modelled treatment pathway.


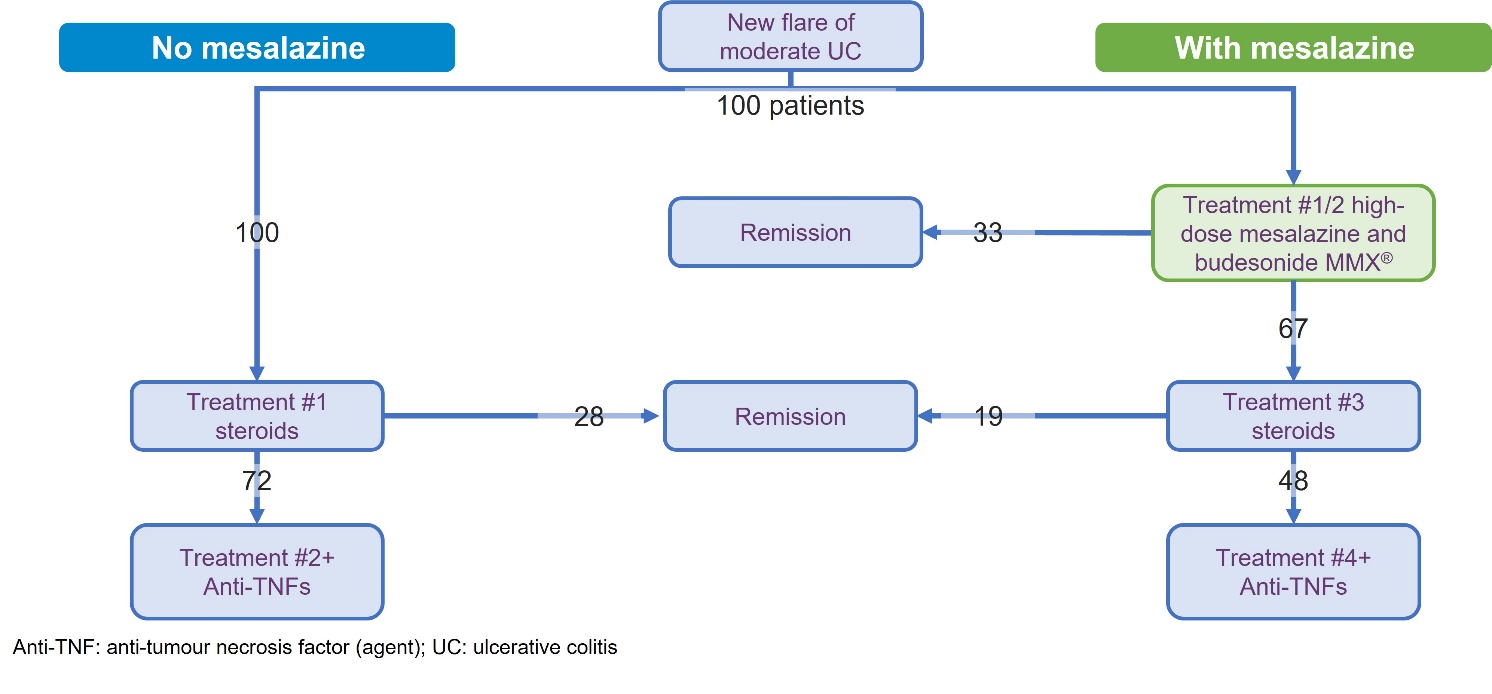


Supplementary Figure 2. Results of model comparing the first-line treatment with and without mesalazine treatment in the induction of remission for moderately active ulcerative colitis for 100 patients.

## Supplementary Tables

Supplementary Table 1. Model results comparing the first-line treatment with and without mesalazine treatment in the induction of remission for moderately active ulcerative colitis split by mesalazine type.

| **Treatment** | **Prolonged-release** | **MMX** | **Eudragit S** |
| --- | --- | --- | --- |
| Patients avoiding steroids | 3,414 | 3,285 | 3,161 |
| Percentage reduction | 34.1% | 32.9% | 31.6% |
| Patients avoiding biologics | 2,455 | 2,362 | 2,272 |
| Percentage reduction | 34.1% | 32.9% | 31.6% |
| Total cost saving | £6,879,176 | £6,484,752 | £6,103,160 |
| Cost saving *per* patient | £687.92 | £648.48 | £610.32 |
